# Gold Nanoclusters as Electrocatalysts for Energy Conversion

**DOI:** 10.3390/nano10020238

**Published:** 2020-01-29

**Authors:** Tokuhisa Kawawaki, Yuichi Negishi

**Affiliations:** 1 Department of Applied Chemistry, Faculty of Science, Tokyo University of Science, 1–3 Kagurazaka, Shinjuku-ku, Tokyo 162–8601, Japan; kawawaki@rs.tus.ac.jp; 2 Photocatalysis International Research Center, Tokyo University of Science, 2641 Yamazaki, Noda, Chiba 278−8510, Japan

**Keywords:** gold, cluster, catalyst, hydrogen evolution reaction, oxygen evolution reaction, oxygen reduction reaction, water splitting, fuel cells, alloy, ligand-protected

## Abstract

Gold nanoclusters (Au*_n_* NCs) exhibit a size-specific electronic structure unlike bulk gold and can therefore be used as catalysts in various reactions. Ligand-protected Au*_n_* NCs can be synthesized with atomic precision, and the geometric structures of many Au*_n_* NCs have been determined by single-crystal X-ray diffraction analysis. In addition, Au*_n_* NCs can be doped with various types of elements. Clarification of the effects of changes to the chemical composition, geometric structure, and associated electronic state on catalytic activity would enable a deep understanding of the active sites and mechanisms in catalytic reactions as well as key factors for high activation. Furthermore, it may be possible to synthesize Au*_n_* NCs with properties that surpass those of conventional catalysts using the obtained design guidelines. With these expectations, catalyst research using Au*_n_* NCs as a model catalyst has been actively conducted in recent years. This review focuses on the application of Au*_n_* NCs as an electrocatalyst and outlines recent research progress.

## 1. Introduction

Gold nanoclusters (Au*_n_* NCs) have physical/chemical properties that differ from those of bulk Au owing to their size-specific electrical/geometrical structure [1,2,3,4,5,6,7,8,9,10,11,12,13,14,15,16,17,18,19,20,21,22]. Therefore, Au*_n_* NCs have been actively studied since the 1960s from the viewpoints of both basic science and application. Since Brust et al., discovered a method for synthesizing Au*_n_* NCs protected by thiolate (Au*_n_*(SR)*_m_*) in 1994 [1], researches on Au*_n_* NCs in particular have grown [6]. Au*_n_*(SR)*_m_* NCs exhibit high stability both in solution and in the solid state because Au forms a strong bond with SR. In addition, Au*_n_*(SR)*_m_* NCs can be synthesized by simply mixing reagents under the ambient atmosphere. Au*_n_*(SR)*_m_* NCs with these unique characteristics have a low handling threshold even for researchers unfamiliar with the chemical synthesis of metal clusters. Au*_n_*(SR)*_m_* NCs are thus currently one of the most studied metal NCs [1,2,3,4,5,6,7,8,9,10,11,12,13,14,15,16,17,18]. For these Au*_n_*(SR)*_m_* NCs, it became possible to synthesize a series of Au*_n_*(SR)*_m_* NCs with atomic precision in 2005 [19]. In addition, since 2007, the geometric structures of many Au*_n_*(SR)*_m_* NCs have been determined through single-crystal X-ray diffraction (SC-XRD) analysis [20]. Since 2009, partial replacement of the Au atoms of Au*_n_*(SR)*_m_* NCs with other elements such as silver (Ag), copper (Cu), platinum (Pt), palladium (Pd), cadmium (Cd), and mercury (Hg) has also been realized [3,4,5,23,24,25,26,27,28,29,30,31,32,33,34,35,36,37,38,39,40,41,42,43,44].

In parallel to these synthesis and structural analysis studies, studies on the functions of Au*_n_* NCs have also been actively conducted. Au*_n_* NCs have been observed to possess catalytic activity for several reactions, including carbon monoxide oxidation [45,46,47,48,49,50,51,52,53,54,55], alcohol oxidation [56,57,58,59,60,61,62,63,64,65], styrene oxidation [66,67,68,69,70], aromatic compound oxidation [71,72], sulfide oxidation [73,74,75], and carbon dioxide reduction [76,77,78,79,80,81,82,83]. One of the reasons for these active studies on the catalysis of Au*_n_* NCs is that their electronic and geometric structures are well understood. Thus, if the obtained catalytic properties are compared with the electronic/geometrical structures of Au*_n_*(SR)*_m_* NCs, information on active sites, mechanisms, and key factors for high activation in catalytic reactions can be obtained. With these expectations, Au*_n_*(SR)*_m_* NCs have received great attention as model catalysts [45,46,47,48,49,50,51,52,53,54,55,56,57,58,59,60,61,62,63,64,65,66,67,68,69,70,71,72,73,74,75,76,77,78,79,80,81,82,83].

**Figure 1:**
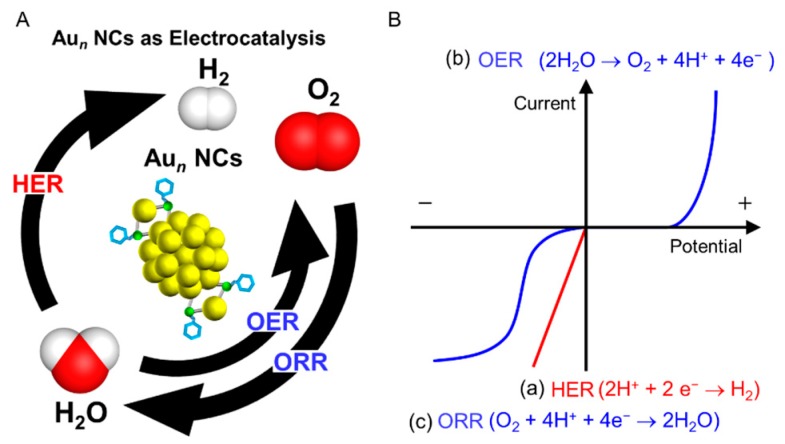
(**A**) Schematic illustration of gold nanoclusters (Au*_n_* NCs) for an electrocatalytic reaction in water splitting (hydrogen evolution reaction (HER) and oxygen evolution reaction (OER)) and fuel cells (oxygen reduction reaction (ORR)). (**B**) Current–potential characteristics for (a) HER, (b) OER, and (c) ORR.

In addition, several studies on Au*_n_*(SR)*_m_* NCs as electrocatalysts have also been performed recently. To prevent serious environmental issues including the depletion of fossil fuels and global warming, the establishment of a system in which hydrogen (H_2_) is generated from water and solar energy using a photocatalyst is desired, with the generated H_2_ used for the generation of electricity using fuel cells [84,85]. Once such an energy conversion system is established, it will be possible to circulate an energy medium (H_2_) in addition to obtaining electricity only from solar energy and abundant water resources. However, realization of such an ultimate energy conversion system requires further improvement of the reaction efficiency of each half reaction of water splitting and fuel cells, including the hydrogen evolution reaction (HER), oxygen evolution reaction (OER), hydrogen oxidation reaction (HOR), and oxygen reduction reaction (ORR; Figure 1A).

To improve the reactivity per unit volume, it is necessary to increase the specific surface area of the active sites and increase the reaction rate at the active sites. For the former, size reduction of the catalyst is one effective method. However, the latter is strongly related to the adsorption energy of reactive molecules on the catalyst surface. The activity of the chemical reaction on the catalyst surface is the highest when the Gibbs energy of adsorption between the catalyst and reactant is moderate according to the Sabatier principle [86]. This is because the reaction does not occur without the adsorption of reactants but is inhibited by the strong adsorption of reactants. Therefore, the relationship between the reaction efficiency and the Gibbs energy for the adsorption of reactants follows a curved line called an activity volcano plot [87]. Fine nanoparticle catalysts suitable for the HER [88,89,90,91,92], OER [93,94,95], and ORR [96,97,98,99,100,101] have been developed based on theoretical predictions of activity volcano plots using various metals and alloy nanoparticles (NPs). Au*_n_* NCs have recently been observed to possess catalytic activity for the HER, OER, and ORR [77,102,103,104,105,106,107,108,109,110,111,112,113,114,115,116] (Figure 1). Therefore, Au*_n_* NCs are expected to become a model catalyst even in such an energy conversion system. A better understanding of the correlation between electronic/geometrical structures and the catalytic activity of the HER, OER, and ORR in Au*_n_* NCs might lead to the discovery of new key factors for achieving high activation. Furthermore, because Au*_n_* NCs are composed of several tens of atoms or less, the use of fine Au*_n_* NCs as a catalyst is also effective in reducing the consumption of expensive noble metals. Thus, it may be possible to create HER, OER, and ORR catalysts with properties that surpass those of conventional catalysts using these unique characteristics of Au*_n_* NCs. With these expectations, several groups are conducting research on the application of Au*_n_* NCs as electrocatalysts. This article reviews the basic theory of electrocatalysts and recent research on HER, OER, and ORR catalysts using Au*_n_* NCs and their alloy NCs.

## 2. Electrocatalytic Reaction in Water Splitting

H_2_ is expected to be an important energy source to support a sustainable energy society. Currently, H_2_ is generated as a by-product during steam reforming or coke production. However, if a water-splitting reaction using an electrocatalyst can be applied for hydrogen production, the large-scale facility of the current system would not be required. In addition, it would be possible to produce H_2_ only with water and electricity using the surplus power from a power plant. Therefore, water electrolysis is considered one of the cleanest energy production reactions for a sustainable energy society.

The water-splitting reaction consists of two half reactions, the HER and OER. When a voltage is applied to the metal electrode, a reduction reaction proceeds at the cathode and an oxidation reaction proceeds at the anode, resulting in the decomposition of water molecules into H_2_ and O_2_ at each electrode. However, the reactions do not proceed even if a potential equal to or higher than both the oxidation and reduction potentials in each reaction (HER: 0 V vs. SHE, OER: 1.23 V vs. SHE; SHE = standard hydrogen electrode) is applied to the electrode. This is because the activation energy of each reaction is too high. Therefore, noble metal NPs are used as a catalyst to reduce the activation energy of the reaction.

### 2.1. Hydrogen Evolution Reaction 

In the HER, metal surface atoms of the catalyst form bonding orbitals with protons (H^+^) through the Volmer–Heyrovsky or Volmer–Tafel mechanism, producing molecular hydrogen [117].

Under acidic conditions, the following reactions occur:

Volmer reaction: M + H^+^ + e^−^ → M–H (1)

Heyrovsky reaction: M–H + H^+^ + e^−^ → M–H_2_ (2)

Tafel reaction: 2M–H → 2M + H_2_ (3)

However, under alkaline conditions, the following reactions occur:

Volmer reaction: 2M + 2H_2_O + 2e^−^ → 2M–H + 2OH^−^ (4)

Heyrovsky reaction: M–H + H_2_O + e^−^ → M–H_2_ + OH^−^ (5)

Tafel reaction: 2M–H → 2M + H_2_ (6)

**Figure 2:**
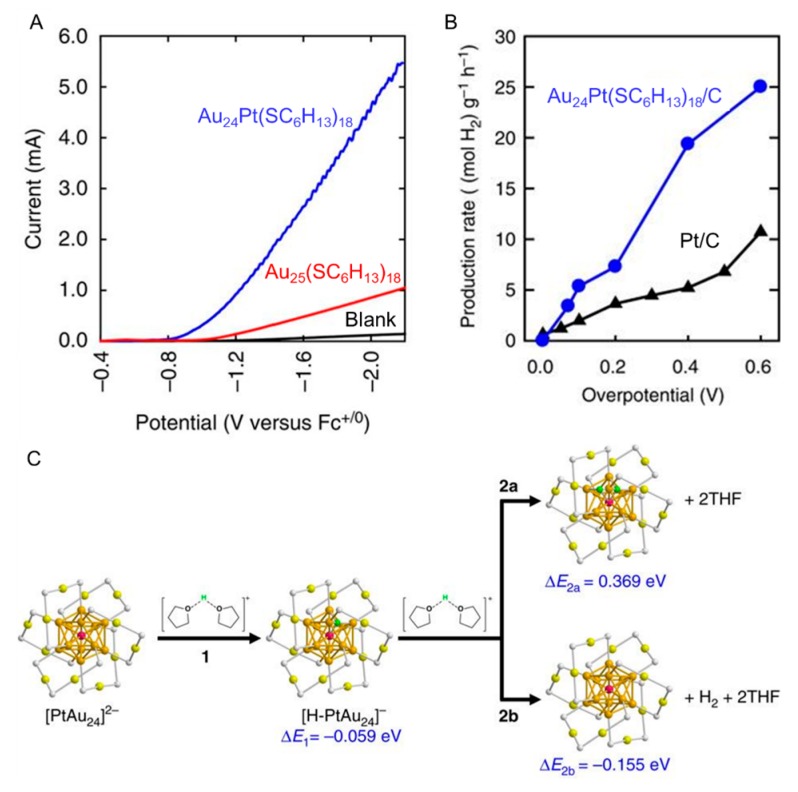
(**A**) HER polarization curves of Au_25_(SC_6_H_13_)_18_- or Au_24_Pt(SC_6_H_13_)_18_-adsorbed glassy carbon electrode (GCE), or GCE. (**B**) H_2_ production rates per mass of metals in the catalyst of Au_24_Pt(SC_6_H_13_)_18_/C (blue circles) and Pt/C (black triangles) electrodes. (**C**) DFT calculation results for Au_24_Pt(SCH_3_)_18_. Color code: golden = Au core; olive = Au shell; purple = Pt; green = adsorbed H from the liquid medium; grey = S. Panels (**A**–**C**) are reproduced with permission from reference [102]. Copyright Springer Nature, 2017.

Bulk Au possesses almost no HER activity, whereas Au*_n_*(SR)*_m_* NCs possess HER activity. In addition, their activity can be further improved by doping Au*_n_*(SR)*_m_* NCs with appropriate heterogeneous elements. These effects were reported by Lee and Jiang et al., in 2017 [102]. They evaluated the HER activity using linear sweep voltammetry (LSV) in tetrahydrofuran (THF) solution with 1.0 M trifluoroacetic acid (TFA) and 0.1 M tetrabutylammonium hexafluorophosphate (Bu_4_NPF_6_) in the absence (black) and presence of Au_25_(SC_6_H_13_)_18_ or Au_24_Pt(SC_6_H_13_)_18_ (SC_6_H_13_ = 1-hexanethiolate) on a glassy carbon electrode (GCE). The onset potential of the HER (Figure 1B(a)) occurred at −1.25 V for the GCE blank (Figure 2A, black line), whereas it occurred at −1.1 V for the GCE with Au_25_(SC_6_H_13_)_18_ (Figure 2A, red line). In addition, for the GCE with Au_24_Pt(SC_6_H_13_)_18_, the onset potential of the HER was further reduced to −0.89 V (Figure 2A, blue line). These findings indicated that Au*_n_*(SR)*_m_* NCs has catalytic activity for the HER and that the HER activity can be further improved by substituting one Au atom of the Au*_n_*(SR)*_m_* NCs with a Pt atom (Table 1). They estimated the HER energies of Au_25_(SCH_3_)_18_ and Au_24_Pt(SCH_3_)_18_ (SCH_3_ = methanethiolate) using density functional theory (DFT) calculations to elucidate the reasons for this behavior (Figure 2C). In these DFT calculations, H^+^ solvated by two THF molecules was used as H^+^. The resulting energy change in the Volmer step was 0.539 eV for [Au_25_(SCH_3_)_18_]^−^, indicating that this reaction is endothermic. However, the energy change in the Volmer step was −0.059 eV for [Au_24_Pt(SCH_3_)_18_]^2−^, indicating that there is almost no energy change (Figure 2C, step 1). The higher HER activity of Au_24_Pt(SC_6_H_13_)_18_ was explained by these differences in the energy barriers in the reaction. In addition, Au_24_Pt(SC_6_H_13_)_18_ possessed higher HER activity even compared with Pt NPs, which are highly active materials for the HER (Figure 2B).

Lee and Jiang et al., observed that a high HER activity and a high catalyst turnover frequency (TOF) can be achieved by doping Au_25_(SC_6_H_13_)_18_ with not only Pt but also Pd (Au_24_Pt(SC_6_H_13_)_18_ > Au_24_Pd(SC_6_H_13_)_18_ > Au_25_(SC_6_H_13_)_18_) [103]. They reported that TOF values of Au_25_(SC_6_H_13_)_18_, Au_24_Pd(SC_6_H_13_)_18_, and Au_24_Pt(SC_6_H_13_)_18_ were 8.2, 13.0, and 33.3 mol H_2_ (mol catalyst)^−1^ s^−1^ at −0.60 V vs. the reversible hydrogen electrode (RHE), respectively. In addition, it was revealed that the doping of Au_38_(SR)_24_ with different elements results in a similar activity enhancement effect with Au_25_(SC_6_H_13_)_18_ (Au_36_Pt_2_(SC_6_H_13_)_24_ > Au_36_Pd_2_(SC_6_H_13_)_24_ > Au_38_(SC_6_H_13_)_24_) [103]. These results are in good agreement with the DFT calculation results. In addition to these studies, Jiang et al., also investigated the doping effects of various elements (Pt, Pd, Ag, Cu, Hg, and Cd) in Au_25_(SCH_3_)_18_ using DFT calculations [105]. The results predicted that Au_24_Pt(SCH_3_)_18_, Au_24_Pd(SCH_3_)_18_, and Au_24_Cu(SCH_3_)_18_, in which the heteroatom (Pt, Pd, or Cu) is located at the center of the metal core, have a higher HER activity than Au_25_(SCH_3_)_18_. Zhu et al., reported that another fine alloy NC, Au_2_Pd_6_(S_4_(PPh_3_)_4_(PhF_2_S)_6_) (PPh_3_ = triphenylphosphine, PhF_2_S = 3,4-difluorobenzenethiolate), also exhibits HER activity (Table 1) [106]. These studies revealed that Au*_n_*(SR)*_m_* and their alloy NCs have HER activity and it can be improved by controlling the electronic structure of Au*_n_* NCs through heteroatom doping.

**Figure 3:**
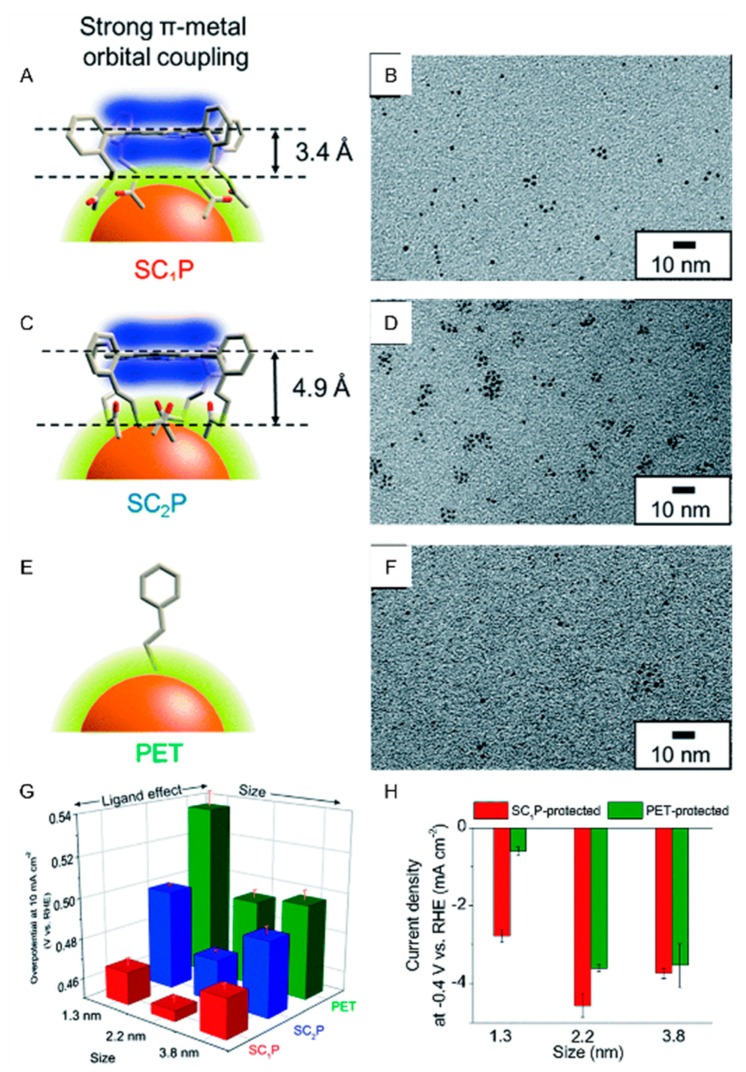
(**A**,**C**,**E**) Schematic illustration of coordination of ligands: (**A**) porphyrin SC_1_P, (**C**) porphyrin SC_2_P, and (**E**) PET. (**B**,**D**,**F**) TEM images of Au NCs with a core size of approximately 1.3 nm protected by porphyrin SC_1_P, porphyrin SC_2_P, or PET, respectively. (**G**) Comparison of overpotential at −10 mA cm^−2^ and (**H**) current density at −0.4 V of each size of Au NCs protected with each ligand. Panels (**A**–**H**) are reproduced with permission from reference [107]. Copyright Royal Society of Chemistry, 2018.

The HER activity varies depending not only on the chemical composition of the metal core but also on the properties of the ligand. In 2018, Teranishi and Sakamoto et al., used Au*_n_* NCs coordinated with SR-containing porphyrin (porphyrin SC*_x_*P). They investigated the effects of the ligand structure on the HER activity of Au*_n_*(SR)*_m_* NCs [107]. In these clusters, the porphyrin ring coordinates horizontally to the gold core. Then, the distance between the porphyrin ring and the Au surface was controlled by changing the length of the alkyl chain between the porphyrin ring and the acetylthio group (Figure 3A,C) [118,119]. The alkyl chain is a methylene chain for porphyrin SC_1_P and an ethylene chain for porphyrin SC_2_P. The distance between the porphyrin ring and the acetylthio group was determined to be 3.4 Å for porphyrin SC_1_P and 4.9 Å for porphyrin SC_2_P by SC-XRD analysis. The researchers synthesized three sizes of Au*_n_* NCs with a core size of approximately 1.3, 2.2, or 3.8 nm using porphyrin SC_1_P, porphyrin SC_2_P, or a common protective ligand, 2-phenylethanethiolate (PET). Transmission electron microscope (TEM) images of the synthesized Au*_n_*(SR)*_m_* NCs (SR = porphyrin SC_1_P, porphyrin SC_2_P, or PET) with a core size of approximately 1.3 nm are presented in Figure 3B,D,F, respectively. Among these products, matrix-assisted laser desorption/ionization mass spectrometry indicated that Au*_n_*(porphyrin SC_1_P)*_m_* NCs consisted of 77 Au atoms and 8 porphyrin SC_1_P molecules and Au*_n_*(porphyrin SC_2_P)*_m_* NCs consisted of 75 Au atoms and 11 porphyrin SC_2_P molecules. The effects of the ligand structure and Au core size on the HER activity of Au*_n_*(SR)*_m_* NCs were investigated using the obtained nine types of Au*_n_*(SR)*_m_* NCs. As a result, in Au*_n_*(SR)*_m_* NCs with a core size of approximately 1.3 nm, Au*_n_*(porphyrin SC_1_P)*_m_* and Au*_n_*(porphyrin SC_2_P)*_m_* NCs exhibited higher current densities of the HER than Au*_n_*(PET)*_m_* NCs (Table 1). For instance, Au*_n_*(porphyrin SC_1_P)*_m_* NCs resulted in a 4.6 times higher current density of the HER than Au*_n_*(PET)*_m_* NCs at −0.4 V vs. RHE. In addition, using Au*_n_*(porphyrin SC_1_P)***_m_*** NCs, the HER occurred at a smaller overvoltage than using Au*_n_*(porphyrin SC_2_P)*_m_* NCs. These results indicate that the HER activity of Au*_n_* NCs depends on the type of ligand and the distance between the ligand and the metal core in Au*_n_* NCs [107]. In this work, the Au*_n_*(SR)*_m_* NCs with a core size of approximately 2.2 nm showed higher catalytic activity than those with a core size of approximately 1.3 nm (Figure 3G,H). This size dependence of the catalytic activity is a little strange considering the surface area of the metal core because a reduction of a core size of Au*_n_*(SR)*_m_* NCs typically leads to the increase in the surface area of Au metal core, which are active sites in HER. The authors have not discussed the details on this point in this paper probably due to the difficulty in precisely estimating the surface area of each Au*_n_*(SR)*_m_* NCs.

**Figure 4:**
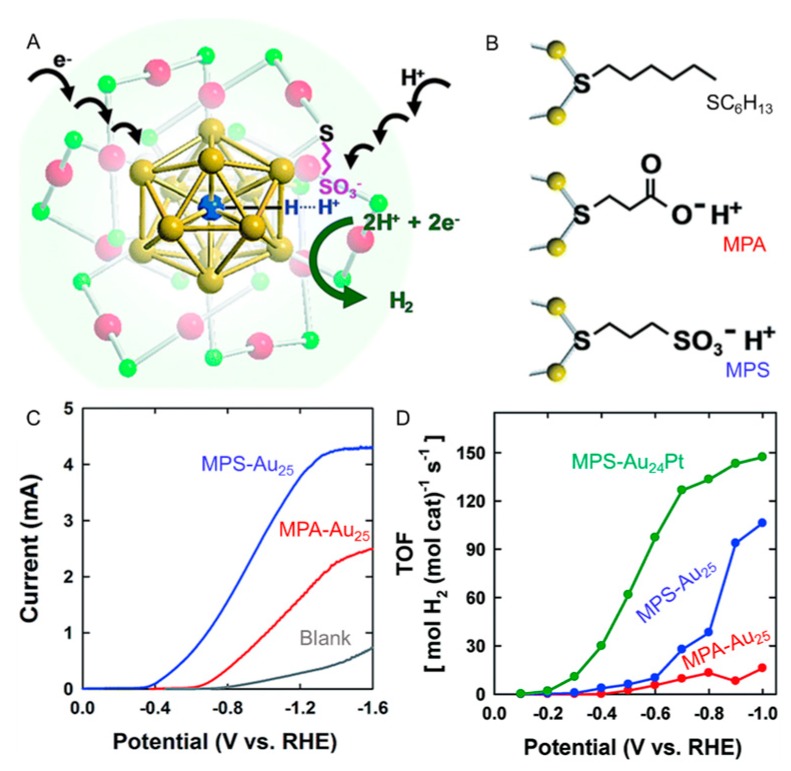
(**A**) Schematic illustration of proton relay mechanism of Au_24_Pt(SR)_18_ nanocluster for formation of H_2_ and (**B**) ligand structures: SC_6_H_13_, MPA, and MPS. Color codes: blue = Pt; golden = core Au; red = shell Au; and green = S. (**C**) HER polarization curves in 0.1 M KCl aqueous solution containing 180 mM acetic acid for MPA-Au_25_ (red) or MPS-Au_25_ (blue). (**D**) turnover frequencies (TOFs) obtained at various potentials in water (3.0 M KCl) containing 180 mM HOAc for MPA-Au_25_ (red), MPS-Au_25_ (blue), or MPS-Au_24_Pt (green). Panels (**A**–**D**) are reproduced with permission from reference [109]. Copyright Royal Society of Chemistry, 2018.

The property of the ligand also strongly affects the interaction between Au*_n_*(SR)*_m_* NCs and the electrode as well as the affinity between Au*_n_*(SR)*_m_* NCs and water molecules. Lee and Jiang et al., synthesized Au*_n_*(SR)*_m_* NCs with SC_6_H_13_, 3-mercaptopropionic acid (MPA), or 3-mercapto-1-propanesulfonic acid (MPS; Figure 4B) as a ligand (Au_25_(SC_6_H_13_)_18_, Au_25_(MPA)_18_, and Au_25_(MPS)_18_) and used them to investigate the effect of ligand properties on the HER activity [109]. In the experiment, Au_25_(SC_6_H_13_)_18_, Au_25_(MPA)_18_, or Au_25_(MPS)_18_ was dissolved at a concentration of 1 mM in 0.1 M KCl aqueous solution, and LSV measurements were performed using a GCE (50 mV s^−1^). Although the blank current was 0.01 mA at −0.7 V vs. RHE (Figure 4C, black line), the HER current of the sample including Au_25_(MPA)_18_ increased up to 0.13 mA at −0.7 V vs. RHE (Figure 4C, red line). When Au_25_(MPS)_18_ was used, a higher HER current of 1.0 mA was observed at −0.7 V vs. RHE (Figure 4C, blue line). MPS and MPA have a hydrophilic functional group (sulfonic acid or carboxylic acid group, respectively) unlike SC_6_H_13_. These hydrophilic functional groups have the property of releasing H^+^ in an aqueous solution. In addition, the sulfonic acid group of MPS (pKa < 1) is expected to have higher H^+^ releasing ability than the carboxylic acid group of MPA (pKa = 3.7). For these reasons, it was interpreted that the difference in the HER activity described above is largely related to the difference in the H^+^ releasing ability of these ligands (Table 1). It was speculated that the energy barrier associated with the intermolecular and intramolecular H^+^ transfer steps is lowered by H^+^ relay in Au*_n_* NCs with high HER activity (Figure 4A). In this paper, they also reported that the use of Au_24_Pt(MPS)_18_, in which Au_25_(MPS)_18_ is replaced with Pt, results in even higher HER activity than Au_25_(MPS)_18_ (Figure 4D and Table 1). They descried that the TOF value of Au_24_Pt(MPS)_18_ was 127 mol H_2_ (mol catalyst)^−1^ s^−1^, which was 4 times higher than that of Au_25_(MPS)_18_ at −0.7 V vs. RHE.

**Figure 5:**
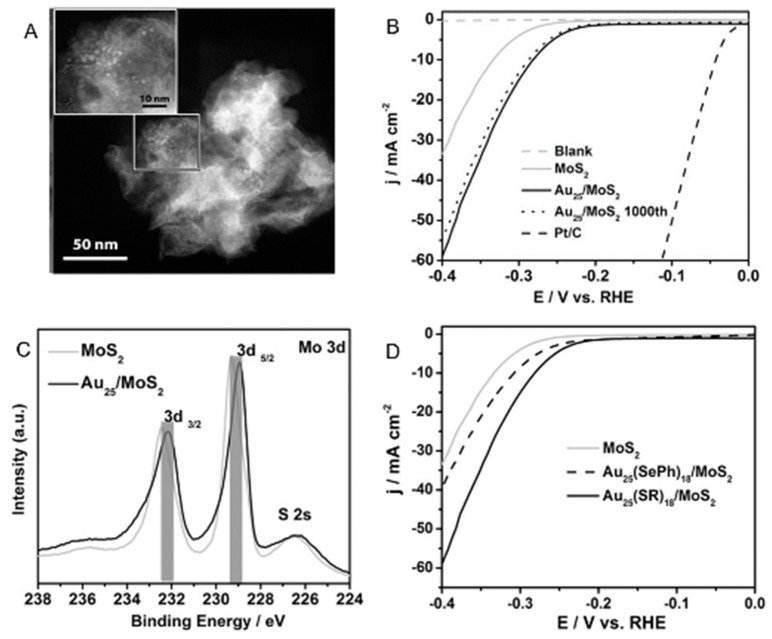
(**A**) High-angle annular dark-field scanning TEM (HAADF-STEM) images, (**B**) HER polarization curves, and (**C**) Mo 3d X-ray photoelectron spectroscopy (XPS) spectra of Au_25_(PET)_18_/MoS_2_. (**D**) HER polarization curves of Au_25_(SePh)_18_/MoS_2_. Panels (**A**–**D**) are reproduced with permission from reference [108]. Copyright Wiley-VCH, 2017.

An electronic interaction also occurs between the Au*_n_*(SR)*_m_* NCs and a catalytic support. This phenomenon was revealed by Jin et al., by measuring the HER activity of MoS_2_ nanosheets (catalytic support) carrying Au_25_(PET)_18_ (Au_25_(PET)_18_/MoS_2_) [108]. In this experiment, Au_25_(PET)_18_/MoS_2_ was prepared by mixing the MoS_2_ nanosheets synthesized by the hydrothermal method and Au_25_(PET)_18_ in dichloromethane for 1 h and drying the obtained products under nitrogen atmosphere. High-angle annular dark-field scanning TEM (HAADF-STEM) images confirmed that Au_25_(PET)_18_ was uniformly supported on MoS_2_ (Figure 5A). Au_25_(PET)_18_/MoS_2_ was then loaded on a GCE, and the HER polarization curve of Au_25_(PET)_18_/MoS_2_ was obtained by scanning the potential in a 0.5 M H_2_SO_4_ aqueous solution using the rotating disk electrode (RDE) method (Figure 5B,D). MoS_2_ without Au_25_(PET)_18_ exhibited a HER overvoltage of 0.33 V at a current density of 10 mA cm^−2^, whereas Au_25_(PET)_18_/MoS_2_ exhibited a smaller HER overvoltage of approximately −0.28 V at the same current density. In addition, Au_25_(PET)_18_/MoS_2_ (59.3 mA cm^−2^) exhibited a 1.79 times higher current density than that of MoS_2_ (33.2 mA cm^−2^) at an applied voltage of −0.4 V vs. RHE. Thus, the HER activity of the MoS_2_ nanosheets was greatly improved by carrying Au_25_(PET)_18_ (Table 1). This improvement of the HER activity was interpreted to be greatly related to the electronic interaction between Au_25_(PET)_18_ and MoS_2_. In fact, X-ray photoelectron spectroscopy (XPS) analysis confirmed that the binding energy of MoS_2_ in the Mo 3 d orbit was negatively shifted by 0.4 eV after Au_25_(PET)_18_ was loaded (Figure 5C). It was assumed that the charge transfer from Au_25_(PET)_18_ to MoS_2_ occurred in Au_25_(PET)_18_/MoS_2_, causing a high HER activity of Au_25_(PET)_18_/MoS_2_. In this study, the HER activity of MoS_2_ nanosheets carrying Au_25_(SePh)_18_ (SePh = phenylselenolate) (Au_25_(SePh)_18_/MoS_2_) was also investigated. Au_25_(SePh)_18_/MoS_2_ was shown to also exhibit higher HER activity than MoS_2_ nanosheets (Table 1). However, the improvement of the activity was smaller than that when carrying Au_25_(PET)_18_ (Figure 5D). This difference was attributed to the difference in the electron interaction and electron relay between Au cores of Au*_n_* NCs and the MoS_2_ nanosheet depending on the ligands. In this way, the HER activity of the Au*_n_* NCs-loaded catalyst was shown to depend on the electronic interaction between the Au*_n_* NCs and the catalytic support.

### 2.2. Oxygen Evolution Reaction 

The OER is a multi-step four-electron reaction in which the reaction proceeds along different reaction paths depending on the binding energy between the metal and the OER intermediate (O, OH, and OOH).

Under acidic conditions, the following reactions occur:

M + H_2_O → M−OH + H^+^ + e^−^ (7)

M−OH → M−O + H^+^ + e^−^ (8)

2(M−O) → 2M + O_2_ (9)

M−O + H_2_O → M−OOH + H^+^ + e^−^ (10)

M−OOH → M + O_2_ + H^+^ + e^−^ (11)

However, under alkaline conditions, the following reactions occur:

M + OH^−^ → M−OH + e^−^ (12)

M−OH + OH^−^ → M−O + H_2_O + e^−^ (13)

2(M−O) → 2M + O_2_ (14)

M−O + OH^−^ → M−OOH + e^−^ (15)

M−OOH + OH^−^ → M + O_2_ + H_2_O + e^−^ (16)

As described above, because the reaction route of OER depends on the intermediates (O, OH, and OOH) on the surface of catalyst, the OER activity of the catalyst also depends on these intermediates. Catalysts that have neither too high nor too low binding energy with oxygen species are suitable for the OER. Previous studies have demonstrated that iridium oxide and ruthenium oxide have such desirable properties. Therefore, miniaturization of these metal oxides and prediction of their physical properties by theoretical calculation have been actively performed [120,121,122,123]. However, because these precious metals are expensive and have the problem of depletion, a search for low-cost catalysts is also being conducted. Related studies have shown that cobalt (Co)-based materials (oxides, hydroxides, selenides, and phosphides) can be used as good OER catalysts. Furthermore, it has been reported that when Au NPs are composited with such Co materials, the OER performance is greatly enhanced as a result of the improved electron conductivity and preferential formation of OOH intermediates on the surface of the catalyst [124,125,126].

**Figure 6:**
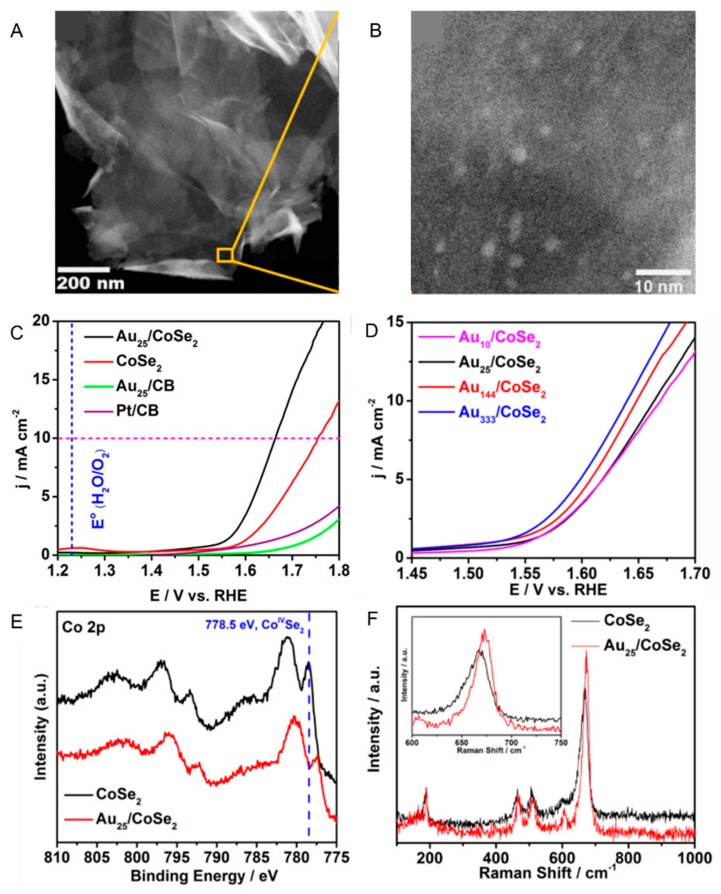
(**A**,**B**) HAADF-STEM images of Au_25_(PET)_18_/CoSe_2_ composite at different magnifications. (**C**,**D**) OER polarization curves of CoSe_2_, Au_10_(SPh-*^t^*Bu)_10_/CoSe_2_, Au_25_(PET)_18_/CoSe_2_, Au_144_(PET)_60_/CoSe_2_, Au_333_(PET)_79_/CoSe_2_, and Pt_NP_/CB (CB = carbon black). (**E**) Co 2p XPS spectra and (**F**) Raman spectra of CoSe_2_ and Au_25_(PET)_18_/CoSe_2_ composite. Panels (**A**–**F**) are reproduced with permission from reference [110]. Copyright American Chemical Society, 2017.

Jin et al., have shown that these mixing effects also occur when Au*_n_* NCs are used instead of Au NPs [110]. In this study, the Au_25_(PET)_18_-loaded CoSe_2_ nanosheet (Au_25_(PET)_18_/CoSe_2_) was prepared by stirring Au_25_(PET)_18_ and CoSe_2_ nanosheets in dichloromethane for 1 h. HAADF-STEM analysis confirmed that Au_25_(PET)_18_ was uniformly supported on the CoSe_2_ nanosheets (Figure 6A,B). Au_25_(PET)_18_/CoSe_2_ was loaded on the GCE, and their OER polarization curves were obtained by scanning the applied potential (5 mV s^−1^) in 0.1 M KOH aqueous solution. The CoSe_2_ nanosheets without Au_25_(PET)_18_ exhibited an OER overvoltage of 0.52 V at a current density of 10 mA cm^−2^ (Figure 1B(b)), whereas Au_25_(PET)_18_/CoSe_2_ exhibited a smaller OER overvoltage of 0.43 V at the same current density (Figure 6C). XPS (Figure 6E) and Raman spectroscopy (Figure 6F) analyses revealed that the electronic interaction occurred between the Au_25_(PET)_18_ and CoSe_2_ nanosheet even in such a composite catalyst. Furthermore, DFT calculation revealed that the formation of the intermediate via OH^−^ is more advantageous by 0.21 eV mol^−1^ at the interface of Co–Au than at the surface of Co. It was thus interpreted that Au_25_(PET)_18_/CoSe_2_ exhibited higher OER activity than the CoSe_2_ nanosheets because Au_25_(PET)_18_/CoSe_2_ stabilized the generation of an OOH intermediate compared with only the CoSe_2_ nanosheet (Table 2). This study also revealed that the OER activity increases with the core size of Au*_n_*(SR)*_m_* NCs (Figure 6D).

## 3. Electrocatalytic Reactions in Fuel Cells

To establish a circulating energy system that does not use fossil fuels and only produces water and a small amount of carbon dioxide as waste, it is essential to further improve the functions of fuel cells. Fuel cells can be roughly classified into those using hydrogen and those using alcohol as a fuel. In fuel cells using hydrogen as a fuel, the HOR and ORR are involved in the system. The HOR is a one-electron reaction, and generally an HER-active catalyst is also useful for the HOR. However, the ORR is a four-electron reaction, and the reaction process is complicated. In addition, the OER is a reaction under oxidizing conditions, whereas the ORR is a reaction under reducing conditions. The surface state of the catalyst and the accompanying binding to the reactants also differ greatly between the OER and ORR. Therefore, catalysts that are active for OER are not necessarily useful for the ORR. Because the ORR is rate-limiting step in a fuel cell, controlling the ORR is important for further development of fuel cells. The ORR pathways under acidic and alkaline conditions are as follows [94].

Under acidic conditions:

O_2_ + 4H^+^ + 4e^−^ → 2H_2_O (17)

O_2_ + 2H^+^ + 2e^−^ → H_2_O_2_ (18)

H_2_O_2_ + 2H^+^ + 2e^−^ → 2H_2_O (19)

Under alkaline conditions:

O_2_ + 2H_2_O + 4e^−^ → 4OH^−^ (20)

O_2_ + H_2_O + 2e^−^ → OOH^−^ + OH^−^ (21)

OOH^−^ + H_2_O + 2e^−^ → 3OH^−^ (22)

Equations (17) and (20) are four-electron reactions, and Equations (18), (19), (21), and (22) are two-electron reactions. For both sets of reactions, the reactions start with the breaking of the O−O bond. The theoretical redox potential is 1.23 V vs. SHE in the direct four-electron path and 0.68 V vs. SHE in the indirect two-electron path. Therefore, a higher energy conversion efficiency can be achieved using the direct four-electron path, and this reaction path is thus more desirable for fuel cells [81]. Although Pt is a useful catalyst for such a reaction pathway, it is expected to be replaced with another metal element because of the high cost of Pt and the resource depletion issue. In addition, synthesis methods of Pt*_n_* NCs in ambient atmosphere with atomic precision are limited, and therefore, it is difficult to study the ORR mechanism using Pt*_n_* NCs as model catalysts. However, for Au*_n_* NCs, there are many examples of synthesis with atomic precision, and these catalysts are stable in ambient atmosphere. In addition, theoretical calculations [127,128] and experimental results [65,129] have predicted that O_2_ molecules can be highly activated on the surface of Au*_n_* NCs. For these reasons, several studies have also been performed on the application of Au*_n_* NCs as ORR catalysts.

**Figure 7:**
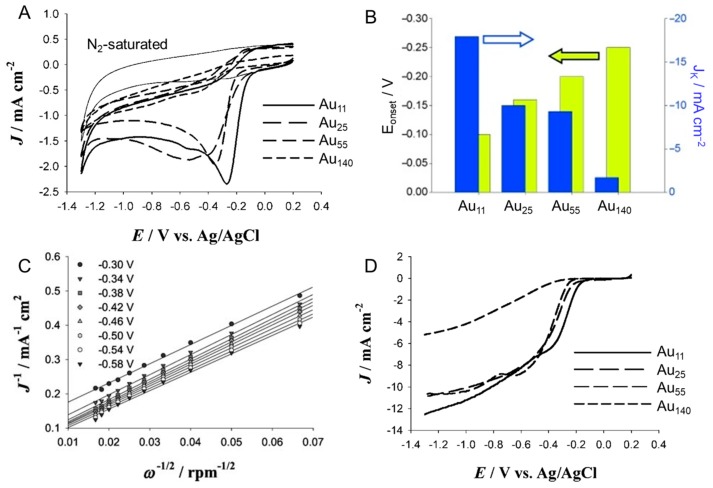
(**A**) Cyclic voltammograms of Au*_n_*(SR)*_m_*/GCE (*n* = 11, 25, 55, and 140) saturated with O_2_ and Au_11_(PPh_3_)_8_Cl_3_/GCE saturated with N_2_ (thin solid curve). (**B**) Current density and overpotential of ORR activity with each size of Au*_n_* NCs. (**C**) Koutecky–Levich plots at different applied potentials of a GCE modified with Au_11_(PPh_3_)_8_Cl_3_. (**D**) Rotating-disk voltammograms (rotation rate: 3600 rpm) of various Au*_n_*(SR)*_m_*/GCE (*n* = 11, 25, 55, and 140). Panels (**A**–**D**) are reproduced with permission from reference [111]. Copyright Wiley-VCH, 2009.

In 2009, Chen et al., evaluated the ORR catalytic activity of Au_11_(PPh_3_)_8_Cl_3_, Au_25_(PET)_18_, Au_55_(PPh_3_)_12_Cl_6_, and Au_140_(SC_6_H_13_)_53_ (Cl = chlorine) [111]. In this experiment, after a series of Au*_n_* NCs were loaded on the GCE, the ORR activity was measured by scanning the potential using the RDE method in a 0.1 M KOH aqueous solution filled with O_2_. When Au_11_(PPh_3_)_8_Cl_3_ was used as the Au*_n_* NCs, the onset potential of the ORR (Figure 1B(c)) was about −0.08 V, and the peak current density was 2.4 mA cm^−2^ (Figure 7A). However, when Au_140_(SC_6_H_13_)_53_ was used as the Au*_n_* NCs, the onset potential shifted to the more cathodic −0.22 V and the reduction peak current decreased to less than 1.0 mA cm^−2^. These results and those for the other two Au*_n_* NCs indicated that the ORR activity increased with decreasing Au core size (Au_11_(PPh_3_)_8_Cl_3_ > Au_25_(PET)_18_ > Au_55_(PPh_3_)_12_Cl_6_ > Au_140_(SC_6_H_13_)_53_) (Figure 7A,B and Table 3). From estimation of the number of electrons for the ORR from a Koutecky–Levich plot [85], it was observed that the relatively small size of Au*_n_* NCs (Au_11_(PPh_3_)_8_Cl_3_, Au_25_(PET)_18_, and Au_55_(PPh_3_)_12_Cl_6_) resulted in the occurrence of the four-electron reaction, whereas Au_140_(SC_6_H_13_)_53_ tended to follow the two-electron reaction pathway (Figure 7C,D). Later, these researchers also synthesized a series of Au*_n_*(SR)*_m_* NCs (Au_25_(PET)_18_, Au_38_(PET)_24_, and Au_144_(PET)_60_) with PET ligands and measured their ORR activities. The results revealed that a smaller core size was associated with higher ORR activity: Au_25_(PET)_18_ > Au_38_(PET)_24_ > Au_144_(PET)_60_ (Table 3) [112]. As the core size decreased, the ratio of low-coordinated surface atoms increased and the d-band center of the Fermi level changed. It was interpreted that smaller Au*_n_*(SR)*_m_* NCs exhibited higher ORR activity because the promotion of oxygen adsorption on the gold core surface was accelerated by miniaturization of the metal core.

**Figure 8:**
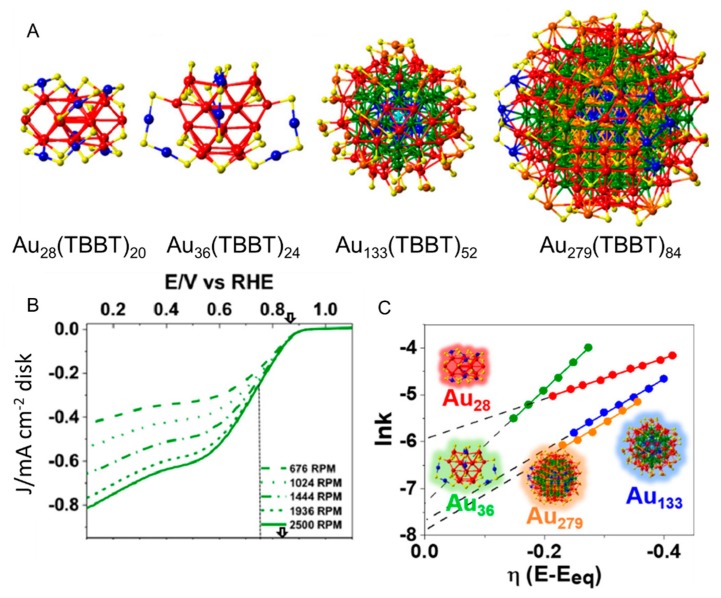
(**A**) X-ray crystal structures of Au*_n_*(TBBT)*_m_* NCs (*n* = 28, 36, 133, and 279). (**B**) Rotating-disk voltammograms recorded for the ORR activity of Au_36_(TBBT)_24_/GCE at different rotation rates. (**C**) Reaction rate constant ln(*k*) vs. overpotential E plots with each size of Au*_n_*(TBBT)*_m_* (*n* = 28, 36, 133, and 279). Panels (**A**–**C**) are reproduced with permission from reference [113]. Copyright American Chemical Society, 2018.

On the other hand, Dass et al., studied the dependence of the ORR activity on the core size using Au*_n_* NCs protected by 4-*tert*-butylbenzenethiolate (TBBT), whose structure differs significantly from that of PET [113]. In this experiment, single-walled carbon nanotubes (SWNTs) carrying Au*_n_*(TBBT)*_m_* NCs (*n* = 28, 36, 133, and 279; Figure 8A; Au*_n_*(TBBT)*_m_* NCs/SWNTs) were loaded onto the GCE. The ORR actives were measured by scanning the potential using the RDE method in a 0.1 M KOH aqueous solution filled with O_2_ (Figure 8B). The overvoltage of the ORR was smaller in the order of Au_36_(TBBT)_24_ > Au_133_(TBBT)_52_ > Au_279_(TBBT)_84_ > Au_28_(TBBT)_20_. However, the selectivity of the four-electron reduction reaction was superior in the order of Au_36_(TBBT)_24_ ≈ Au_133_(TBBT)_52_ > Au_279_(TBBT)_84_ > Au_28_(TBBT)_20_ [113] (Figure 8C). Notably, this trend was similar to that of the size dependence of the stability of Au*_n_*(TBBT)*_m_* NCs itself. The same group performed similar studies using *tert*-butylthiolate (S-*^t^*Bu) instead of TBBT as a ligand [114]. S-*^t^*Bu has a bulky framework and when this ligand is used in the synthesis of Au*_n_*(SR)*_m_* NCs, the ratio of the metal atom and the ligand in the generated Au*_n_*(SR)*_m_* NCs is different from that in Au*_n_*(SR)*_m_* NCs synthesized using another ligand. Such Au*_n_*(S-*^t^*Bu)*_m_* NCs exhibit a unique size dependency for ORR activity (Au_65_(S-*^t^*Bu)_29_ > Au_46_(S-*^t^*Bu)_24_ > Au_30_(S-*^t^*Bu)_18_ > Au_23_(S-*^t^*Bu)_16_) [114].

**Figure 9:**
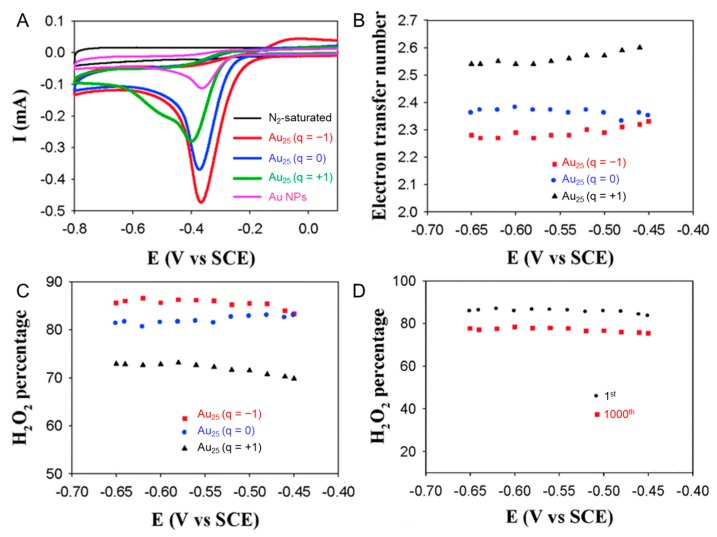
(**A**) Cyclic voltammograms, (**B**) electron transfer number (*n*), and (**C**) percentage of H_2_O_2_ of the ORR on Au_25_(SC_12_H_25_)_18_ with different charge states ([Au_25_(SC_12_H_25_)_18_]^−^, [Au_25_(SC_12_H_25_)_18_]^0^, and [Au_25_(SC_12_H_25_)_18_]^+^) in 0.1 M KOH aq saturated with O_2_. (**D**) Accelerated durability tests of [Au_25_(SC_12_H_25_)_18_]^−^ performed for 1000 cycles. Panels (**A**–**D**) are reproduced with permission from reference [115]. Copyright Royal Society of Chemistry, 2014.

In addition to these effects of core sizes and ligands, the ORR activity also depended on the charge state of Au*_n_*(SR)*_m_* NCs. Chen et al., carried [Au_25_(SC_12_H_25_)_18_]^−^, [Au_25_(SC_12_H_25_)_18_]^0^, and [Au_25_(SC_12_H_25_)_18_]^+^ (SC_12_H_25_ = 1-dodecanethiolate) on the GCE, and their ORR activities were evaluated by scanning the potential in a 0.1 M KOH aqueous solution using a rotating ring-disk electrode (RRDE) filled with O_2_ [115]. In addition, the generation of H_2_O_2_ was evaluated from the RRDE current at a fixed ring potential (0.5 V vs. saturated calomel electrode (SCE)). When [Au_25_(SC_12_H_25_)_18_]^−^, [Au_25_(SC_12_H_25_)_18_]^0^, and [Au_25_(SC_12_H_25_)_18_]^+^ were used, the efficiencies of H_2_O_2_ were 86%, 82%, and 72%, respectively. In addition, the number of electrons for the ORR was estimated to be 2.28 ([Au_25_(SC_12_H_25_)_18_]^−^), 2.35 ([Au_25_(SC_12_H_25_)_18_]^0^), and 2.56 ([Au_25_(SC_12_H_25_)_18_]^+^; Figure 9A–C). For [Au_25_(SC_12_H_25_)_18_]^−^, which showed the highest production rate of H_2_O_2_, the activity decreased only 9% even after 1000 cycles (Figure 9D). These results indicate that [Au_25_(SC_12_H_25_)_18_]^−^ has high H_2_O_2_ generating ability (Table 3) [115]. Since H_2_O_2_ is a useful raw material for chemical products, the development of their highly selective production reactions is important. Jin et al., also studied the dependence of the ORR activity on the charge state of Au*_n_*(SR)*_m_* NCs using [Au_25_(PET)_18_]^−^, [Au_25_(PET)_18_]^0^, and [Au_25_(PET)_18_]^+^. They reported that too strong of an OH^−^ adsorbing ability of [Au_25_(PET)_18_]^+^ reduces the ORR activity [77]. Thus, it has been clarified that the charge state of Au*_n_*(SR)*_m_* NCs also has a significant effect on the ORR activity of Au*_n_*(SR)*_m_* NCs.

## 4. Conclusions 

A system for the generation of a fuel such as hydrogen or methanol using natural energy (e.g., solar cells or photocatalytic water splitting) and the production of electricity by fuel cells using these fuels would be one of the ultimate energy conversion systems for our society. To realize such a system, high activation of the HER, OER, HOR, and ORR is indispensable. Recently, Au*_n_* NCs have attracted considerable attention as model catalysts for these reactions. In this review, recent works on these materials were summarized. The overall characteristics of the HER, OER, and ORR can be summarized as follows.

1) Since the core size, doping metal, ligand structure, and charge state affect the electronic and geometrical structures of Au*_n_* NCs, these parameters also have a great effect on the catalytic activity of Au*_n_* NCs.

2) Although these three reactions proceed via different mechanisms, reducing the core size of Au*_n_* NCs and improving the ligand conductivity tend to improve the activities.

3) When Au*_n_* NCs are carried on a conventional catalytic support, their electronic structure changes and thus their catalytic activity also changes. Therefore, Au*_n_* NCs are also useful for improving the catalytic activity of conventional catalytic materials.

## 5. Perspectives

Until recently, the materials with relatively high activity for all of HER, OER, and ORR are considered to be limited to Ir, Rh, Ru, and Pt [84,85]. However, the recent studies demonstrated that these properties could also be caused in Au by the discretization of the band structure (e.g., shift of d-band center [107,111]). For Au*_n_* NCs, it is possible to precisely control the electronic/geometrical structures and thereby to elucidate the correlation between catalytic activity and electronic/geometrical structure. In addition, the use of fine Au*_n_* NCs as a catalyst is effective in reducing the consumption of expensive noble metals. It is expected that the studies on the catalytic activities of Au*_n_* NCs lead to solve the mechanism in catalytic reactions on the metal surface and create the amazing catalysts we have never seen.

However, to create such HER, OER, and ORR catalysts using Au*_n_* NCs and their alloy NCs, further studies are required. Previous studies have shown that doping with Group 10 elements (Pt and Pd) induces high activation. Thus, a method for increasing the doping concentration of these elements is expected to be developed in the future. In addition, regarding the HER and OER, in spite of decomposing water, most studies thus far have used hydrophobic ligands that are not compatible with water. This may be related to the fact that the synthesis of hydrophobic Au*_n_* NCs is easier than that of hydrophilic Au*_n_* NCs. In particular, it is difficult to selectively synthesize a group-10-element-doped cluster using a hydrophilic ligand using the conventional synthesis method. However, as shown in this review, it is more appropriate to use hydrophilic Au*_n_* NCs as HER and OER catalysts. Therefore, in the future, additional research on hydrophilic Au*_n_* NCs is expected to increase the types of ligands and core sizes of hydrophilic Au*_n_* NCs. Such studies are expected to lead to the creation of highly active HER, OER, and ORR catalysts and eventually to the development of design guidelines for establishing ultimate energy conversion systems.

## Figures and Tables

**Table 1:** Representative references on HER activity of Au*_n_* NCs and related alloy NCs.

Ligand	Support	Experimental Condition	Activity	Reference
SC_6_H_13_	−	1.0 M TFA and 0.1 M Bu_4_NPF_6_ in THF *^c^*	Au_24_Pt(SC_6_H_13_)_18_ > Au_25_(SC_6_H_13_)_18_	[102]
SC_6_H_13_	carbon black	1 M Britton–Robinson buffer solution in 2 M KCl aq (pH 3) *^c,d^*	Au_24_Pt(SC_6_H_13_)_18_ > Au_24_Pd(SC_6_H_13_)_18_ > Au_25_(SC_6_H_13_)_18_	[103]
SC_6_H_13_	carbon black	1 M Britton–Robinson buffer solution in 2 M KCl aq (pH 3) *^c,d^*	Au_36_Pt_2_(SC_6_H_13_)_24_ > Au_36_Pd_2_(SC_6_H_13_)_24_ > Au_38_(SC_6_H_13_)_24_	[103]
PPh_3_ PPh_2_ *^a^* Cl *^b^* PhF_2_S	MoS_2_	0.5 M phosphate buffer solution (pH 6.7) *^c,d^*	Au_2_Pd_6_(S_4_(PPh_3_)_4_(PhF_2_S)_6_)/MoS_2_ > Mixture of Au_2_Cl_2_C(PPh_2_)_2_ and Pd_3_(Cl(PPh_2_)_2_(PPh_3_)_3_)/MoS_2_ > Pd_3_(Cl(PPh_2_)_2_(PPh_3_)_3_)/MoS_2_ > Au_2_Cl_2_C(PPh_2_)_2_/MoS_2_ > MoS_2_	[104]
porphyrin SC_1_P porphyrin SC_2_P PET	−	0.5 M H_2_SO_4_ aq *^e^*	Au(1.3 nm)(porphyrin SC_1_P) > Au(1.3 nm)(porphyrin SC_2_P) > Au(1.3 nm)(PET)	[107]
PET SePh	MoS_2_	0.5 M H_2_SO_4_ aq *^c,d^*	Au_25_(PET)_18_/MoS_2_ > Au_25_(SePh)_18_/MoS_2_ > MoS_2_	[108]
SC_6_H_13_ MPA MPS	−	0.1 M KCl aq *^c^*	Au_24_Pt(MPS)_18_ > Au_25_(MPS)_18_ > Au_25_(MPA)_18_ > Au_25_(SC_6_H_13_)_18_	[109]

***^a^*** Diphenylphosphine. *^b^* Chlorine. *^c^* WE: Working electrode; GCE. *^d^* WE: Containing Nafion. *^e^* WE: Carbon tape.

**Table 2:** Representative reference on OER activity of Au*_n_*(SR)*_m_* NCs.

Ligand	Support	Experimental Condition	Activity	Reference
PET	CoSe_2_	0.l M KOH aq *^a, b^*	Au_25_(PET)_18_/CoSe_2_ > CoSe_2_	[110]

***^a^*** WE: GCE. *^b^* WE: Containing Nafion.

**Table 3:** Representative references on ORR activity of Au*_n_* NCs.

Ligand	Support	Experimental Condition	Activity	Reference
PET SC_6_H_13_ Cl PPh_3_	−	0.1 M KOH aq *^a^*	Au_11_(PPh_3_)_8_Cl_3_ > Au_25_(PET)_18_ > Au_55_(PPh_3_)_12_Cl_6_ > Au_140_(SC_6_H_13_)_53_	[101]
PET	Reduced graphene oxide	0.1 M KOH aq *^a, b^*	Au_25_(PET)_18_ > Au_38_(PET)_24_ > Au_144_(PET)_60_	[112]
TBBT	SWNTs	0.1 M KOH aq *^a, b^*	Au_36_(TBBT)_24_ > Au_133_(TBBT)_52_ > Au_279_(TBBT)_84_ > Au_28_(TBBT)_20_	[113]
S-*^t^*Bu	SWNTs	0.1 M KOH aq *^a, b^*	Au_65_(S-*^t^*Bu)_29_ > Au_46_(S-*^t^*Bu)_24_ > Au_30_(S-*^t^*Bu)_18_ > Au_23_(S-*^t^*Bu)_16_	[114]
SC_12_H_25_	−	0.1 M KOH aq *^a, b^*	[Au_25_(SC_12_H_25_)_18_]^−^ > [Au_25_(SC_12_H_25_)_18_]^0^ > [Au_25_(SC_12_H_25_)_18_]^+ *c*^	[115]

*^a^* WE: GCE. *^b^* WE; Containing Nafion. *^c^* Tow-electron reduction.

## References

[1] Brust M., Walker M., Bethell D., Schiffrin D.J., Whyman R. (1994). Synthesis of Thiol-Derivatised Gold Nanoparticles in a Two-Phase Liquid–Liquid System. J. Chem. Soc. Chem. Commun..

[2] Jin R., Zeng C., Zhou M., Chen Y. (2016). Atomically Precise Colloidal Metal Nanoclusters and Nanoparticles: Fundamentals and Opportunities. Chem. Rev..

[3] Kurashige W., Niihori Y., Sharma S., Negishi Y. (2016). Precise Synthesis, Functionalization and Application of Thiolate-Protected Gold Clusters. Coord. Chem. Rev..

[4] Hossain S., Niihori Y., Nair L.V., Kumar B., Kurashige W., Negishi Y. (2018). Alloy Clusters: Precise Synthesis and Mixing Effects. Acc. Chem. Res..

[5] Gan Z., Xia N., Wu Z. (2018). Discovery, Mechanism; Application of Antigalvanic Reaction. Acc. Chem. Res..

[6] Chakraborty I., Pradeep T. (2017). Atomically Precise Clusters of Noble Metals: Emerging Link between Atoms and Nanoparticles. Chem. Rev..

[7] Yao Q., Chen T., Yuan X., Xie J. (2018). Toward Total Synthesis of Thiolate-Protected Metal Nanoclusters. Acc. Chem. Res..

[8] Qian H., Zhu M., Wu Z., Jin R. (2012). Quantum Sized Gold Nanoclusters with Atomic Precision. Acc. Chem. Res..

[9] Whetten R.L., Weissker H.-C., Pelayo J.J., Mullins S.M., López-Lozano X., Garzón I.L. (2019). Chiral-Icosahedral (*I*) Symmetry in Ubiquitous Metallic Cluster Compounds (145A,60X): Structure and Bonding Principles. Acc. Chem. Res..

[10] Aikens C.M. (2018). Electronic and Geometric Structure, Optical Properties, and Excited State Behavior in Atomically Precise Thiolate-Stabilized Noble Metal Nanoclusters. Acc. Chem. Res..

[11] Nieto-Ortega B., Bürgi T. (2018). Vibrational Properties of Thiolate-Protected Gold Nanoclusters. Acc. Chem. Res..

[12] Agrachev M., Ruzzi M., Venzo A., Maran F. (2019). Nuclear and Electron Magnetic Resonance Spectroscopies of Atomically Precise Gold Nanoclusters. Acc. Chem. Res..

[13] Pei Y., Wang P., Ma Z., Xiong L. (2019). Growth-Rule-Guided Structural Exploration of Thiolate-Protected Gold Nanoclusters. Acc. Chem. Res..

[14] Ghosh A., Mohammed O.F., Bakr O.M. (2018). Atomic-Level Doping of Metal Clusters. Acc. Chem. Res..

[15] Bigioni T.P., Whetten R.L., Dag Ö. (2000). Near-Infrared Luminescence from Small Gold Nanocrystals. J. Phys. Chem. B.

[16] Yan J., Teo B.K., Zheng N. (2018). Surface Chemistry of Atomically Precise Coinage–Metal Nanoclusters: From Structural Control to Surface Reactivity and Catalysis. Acc. Chem. Res..

[17] Sakthivel N.A., Dass A. (2018). Aromatic Thiolate-Protected Series of Gold Nanomolecules and a Contrary Structural Trend in Size Evolution. Acc. Chem. Res..

[18] Tang Q., Hu G., Fung V., Jiang D.-E. (2018). Insights into Interfaces, Stability, Electronic Properties, and Catalytic Activities of Atomically Precise Metal Nanoclusters from First Principles. Acc. Chem. Res..

[19] Negishi Y., Nobusada K., Tsukuda T. (2005). Glutathione-Protected Gold Clusters Revisited:  Bridging the Gap between Gold(I)−Thiolate Complexes and Thiolate-Protected Gold Nanocrystals. J. Am. Chem. Soc..

[20] Jadzinsky P.D., Calero G., Ackerson C.J., Bushnell D.A., Kornberg R.D. (2007). Structure of a Thiol Monolayer-Protected Gold Nanoparticle at 1.1 Å Resolution. Science.

[21] Wang S., Li Q., Kang X., Zhu M. (2018). Customizing the Structure, Composition, and Properties of Alloy Nanoclusters by Metal Exchange. Acc. Chem. Res..

[22] Higaki T., Li Q., Zhou M., Zhao S., Li Y., Li S., Jin R. (2018). Toward the Tailoring Chemistry of Metal Nanoclusters for Enhancing Functionalities. Acc. Chem. Res..

[23] Negishi Y., Kurashige W., Niihori Y., Iwasa T., Nobusada K. (2010). Isolation, Structure, and Stability of a Dodecanethiolate-Protected Pd_1_Au_24_ Cluster. Phys. Chem. Chem. Phys..

[24] Negishi Y., Iwai T., Ide M. (2010). Continuous Modulation of Electronic Structure of Stable Thiolate-Protected Au_25_ Cluster by Ag Doping. Chem. Commun..

[25] Negishi Y., Igarashi K., Munakata K., Ohgake W., Nobusada K. (2012). Palladium Doping of Magic Gold Cluster Au_38_(SC_2_H_4_Ph)_24_: Formation of Pd_2_Au_36_(SC_2_H_4_Ph)_24_ with Higher Stability than Au_38_(SC_2_H_4_Ph)_24_. Chem. Commun..

[26] Negishi Y., Munakata K., Ohgake W., Nobusada K. (2012). Effect of Copper Doping on Electronic Structure, Geometric Structure, and Stability of Thiolate-Protected Au_25_ Nanoclusters. J. Phys. Chem. Lett..

[27] Niihori Y., Kurashige W., Matsuzaki M., Negishi Y. (2013). Remarkable Enhancement in Ligand-Exchange Reactivity of Thiolate-Protected Au_25_ Nanoclusters by Single Pd Atom Doping. Nanoscale.

[28] Negishi Y., Kurashige W., Kobayashi Y., Yamazoe S., Kojima N., Seto M., Tsukuda T. (2013). Formation of a Pd@Au_12_ Superatomic Core in Au_24_Pd_1_(SC_12_H_25_)_18_ Probed by ^197^Au Mössbauer and Pd K-edge EXAFS Spectroscopy. J. Phys. Chem. Lett..

[29] Negishi Y., Kurashige W., Niihori Y., Nobusada K. (2013). Toward the Creation of Stable, Functionalized Metal Clusters. Phys. Chem. Chem. Phys..

[30] Niihori Y., Matsuzaki M., Uchida C., Negishi Y. (2014). Advanced Use of High-Performance Liquid Chromatography for Synthesis of Controlled Metal Clusters. Nanoscale.

[31] Yamazoe S., Kurashige W., Nobusada K., Negishi Y., Tsukuda T. (2014). Preferential Location of Coinage Metal Dopants (M = Ag or Cu) in [Au_25-*x*_M*_x_*(SC_2_H_4_Ph)_18_]^−^ (*x* ~ 1) as Determined by Extended X-ray Absorption Fine Structure and Density Functional Theory Calculations. J. Phys. Chem. C.

[32] Sharma S., Kurashige W., Nobusada K., Negishi Y. (2015). Effect of Trimetallization in Thiolate-Protected Au_24−*n*_Cu*_n_*Pd Clusters. Nanoscale.

[33] Niihori Y., Eguro M., Kato A., Sharma S., Kumar B., Kurashige W., Nobusada K., Negishi Y. (2016). Improvements in the Ligand-Exchange Reactivity of Phenylethanethiolate-Protected Au_25_ Nanocluster by Ag or Cu Incorporation. J. Phys. Chem. C.

[34] Niihori Y., Hossain S., Kumar B., Nair L.V., Kurashige W., Negishi Y. (2017). Perspective: Exchange Reactions in Thiolate-Protected Metal Clusters. APL Mater..

[35] Niihori Y., Hossain S., Sharma S., Kumar B., Kurashige W., Negishi Y. (2017). Understanding and Practical Use of Ligand and Metal Exchange Reactions in Thiolate-Protected Metal Clusters to Synthesize Controlled Metal Clusters. Chem. Rec..

[36] Niihori Y., Shima D., Yoshida K., Hamada K., Nair L.V., Hossain S., Kurashige W., Negishi Y. (2018). High-Performance Liquid Chromatography Mass Spectrometry of Gold and Alloy Clusters Protected by Hydrophilic Thiolates. Nanoscale.

[37] Hossain S., Ono T., Yoshioka M., Hu G., Hosoi M., Chen Z., Nair L.V., Niihori Y., Kurashige W., Jiang D.-E. (2018). Thiolate-Protected Trimetallic Au_~20_Ag_~4_Pd and Au_~20_Ag_~4_Pt Alloy Clusters with Controlled Chemical Composition and Metal Positions. J. Phys. Chem. Lett..

[38] Yokoyama T., Hirata N., Tsunoyama H., Negishi Y., Nakajima A. (2018). Characterization of Floating-gate Memory Device with Thiolate-Protected Gold and Gold-Palladium Nanoclusters. AIP Adv..

[39] Niihori Y., Koyama Y., Watanabe S., Hashimoto S., Hossain S., Nair L.V., Kumar B., Kurashige W., Negishi Y. (2018). Atomic and Isomeric Separation of Thiolate-Protected Alloy Clusters. J. Phys. Chem. Lett..

[40] Niihori Y., Hashimoto S., Koyama Y., Hossain S., Kurashige W., Negishi Y. (2019). Dynamic Behavior of Thiolate-Protected Gold–Silver 38-Atom Alloy Clusters in Solution. J. Phys. Chem. C.

[41] Kurashige W., Hayashi R., Wakamatsu K., Kataoka Y., Hossain S., Iwase A., Kudo A., Yamazoe S., Negishi Y. (2019). Atomic-Level Understanding of the Effect of Heteroatom Doping of the Cocatalyst on Water-Splitting Activity in AuPd or AuPt Alloy Cluster-Loaded BaLa_4_Ti_4_O_15_. ACS Appl. Energy Mater..

[42] Hossain S., Imai Y., Suzuki D., Choi W., Chen Z., Suzuki T., Yoshioka M., Kawawaki T., Lee D., Negishi Y. (2019). Elucidating Ligand Effects in Thiolate-Protected Metal Clusters Using Au_24_Pt(TBBT)_18_ as a Model Cluster. Nanoscale.

[43] Kawawaki T., Negishi Y., Kawasaki H. (2020). Photo/electrocatalysis and Photosensitization Using Metal Nanoclusters for Green Energy and Medical Applications. Nanoscale Adv..

[44] Hossain S., Imai Y., Motohashi Y., Chen Z., Suzuki D., Suzuki T., Kataoka Y., Hirata M., Ono T., Kurashige W. Understanding and Designing One-Dimensional Assemblies of Ligand-Protected Metal Nanoclusters. Mater. Horiz..

[45] Haruta M. (2011). Spiers Memorial Lecture Role of Perimeter Interfaces in Catalysis by Gold Nanoparticles. Faraday Discuss..

[46] Nie X., Qian H., Ge Q., Xu H., Jin R. (2012). CO Oxidation Catalyzed by Oxide-Supported Au_25_(SR)_18_ Nanoclusters and Identification of Perimeter Sites as Active Centers. ACS Nano.

[47] Nie X., Zeng C., Ma X., Qian H., Ge Q., Xu H., Jin R. (2013). CeO_2_-Supported Au_38_(SR)_24_ Nanocluster Catalysts for CO Oxidation: A Comparison of Ligand-on and -off Catalysts. Nanoscale.

[48] Wu Z., Jiang D.-e., Mann A.K.P., Mullins D.R., Qiao Z.-A., Allard L.F., Zeng C., Jin R., Overbury S.H. (2014). Thiolate Ligands as a Double-Edged Sword for CO Oxidation on CeO_2_ Supported Au_25_(SCH_2_CH_2_Ph)_18_ Nanoclusters. J. Am. Chem. Soc..

[49] Li W., Ge Q., Ma X., Chen Y., Zhu M., Xu H., Jin R. (2016). Mild Activation of CeO_2_-Supported Gold Nanoclusters and Insight into the Catalytic Behavior in CO Oxidation. Nanoscale.

[50] Gaur S., Miller J.T., Stellwagen D., Sanampudi A., Kumar C.S.S.R., Spivey J.J. (2012). Synthesis, Characterization, and Testing of Supported Au Catalysts Prepared from Atomically-Tailored Au_38_(SC_12_H_25_)_24_ Clusters. Phys. Chem. Chem. Phys..

[51] Wu Z., Hu G., Jiang D.-e., Mullins D.R., Zhang Q.-F., Allard L.F., Wang L.-S., Overbury S.H. (2016). Diphosphine-Protected Au_22_ Nanoclusters on Oxide Supports Are Active for Gas-Phase Catalysis without Ligand Removal. Nano Lett..

[52] Wu Z., Mullins D.R., Allard L.F., Zhang Q., Wang L. (2018). CO Oxidation over Ceria Supported Au_22_ Nanoclusters: Shape Effect of the Support. Chin. Chem. Lett..

[53] Lin J., Li W., Liu C., Huang P., Zhu M., Ge Q., Li G. (2015). One-Phase Controlled Synthesis of Au_25_ Nanospheres and Nanorods from 1.3 nm Au : PPh_3_ Nanoparticles: The Ligand Effects. Nanoscale.

[54] Li W., Liu C., Abroshan H., Ge Q., Yang X., Xu H., Li G. (2016). Catalytic CO Oxidation Using Bimetallic M*_X_*Au*_25–X_* Clusters: A Combined Experimental and Computational Study on Doping Effects. J. Phys. Chem. C.

[55] Good J., Duchesne P.N., Zhang P., Koshut W., Zhou M., Jin R. (2017). On the Functional Role of the Cerium Oxide Support in the Au_38_(SR)_24_/CeO_2_ Catalyst for CO Oxidation. Catal. Today.

[56] Du Y., Sheng H., Astruc D., Zhu M. (2020). Atomically Precise Noble Metal Nanoclusters as Efficient Catalysts: A Bridge between Structure and Properties. Chem. Rev..

[57] Xie S., Tsunoyama H., Kurashige W., Negishi Y., Tsukuda T. (2012). Enhancement in Aerobic Alcohol Oxidation Catalysis of Au_25_ Clusters by Single Pd Atom Doping. ACS Catal..

[58] Yoskamtorn T., Yamazoe S., Takahata R., Nishigaki J.-i., Thivasasith A., Limtrakul J., Tsukuda T. (2014). Thiolate-Mediated Selectivity Control in Aerobic Alcohol Oxidation by Porous Carbon-Supported Au_25_ Clusters. ACS Catal..

[59] Lavenn C., Demessence A., Tuel A. (2015). Au_25_(SPh-*p*NH_2_)_17_ Nanoclusters Deposited on SBA-15 as Catalysts for Aerobic Benzyl Alcohol Oxidation. J. Catal..

[60] Deng H., Wang S., Jin S., Yang S., Xu Y., Liu L., Xiang J., Hu D., Zhu M. (2015). Active Metal (Cadmium) Doping Enhanced the Stability of Inert Metal (Gold) Nanocluster under O_2_ Atmosphere and the Catalysis Activity of Benzyl Alcohol Oxidation. Gold Bull..

[61] Li L., Dou L., Zhang H. (2014). Layered Double Hydroxide Supported Gold Nanoclusters by Glutathione-Capped Au Nanoclusters Precursor Method for Highly Efficient Aerobic Oxidation of Alcohols. Nanoscale.

[62] Wang S., Yin S., Chen G., Li L., Zhang H. (2016). Nearly Atomic Precise Gold Nanoclusters on Nickel-Based Layered Double Hydroxides for Extraordinarily Efficient Aerobic Oxidation of Alcohols. Catal. Sci. Technol..

[63] Yin S., Li J., Zhang H. (2016). Hierarchical Hollow Nanostructured Core@Shell Recyclable Catalysts γ-Fe_2_O_3_@LDH@Au_25-*X*_ for Highly Efficient Alcohol Oxidation. Green Chem..

[64] Lee K.E., Shivhare A., Hu Y., Scott R.W.J. (2017). Supported Bimetallic AuPd Clusters Using Activated Au_25_ Clusters. Catal. Today.

[65] Tsunoyama H., Ichikuni N., Sakurai H., Tsukuda T. (2009). Effect of Electronic Structures of Au Clusters Stabilized by Poly(*N*-vinyl-2-pyrrolidone) on Aerobic Oxidation Catalysis. J. Am. Chem. Soc..

[66] Zhu Y., Qian H., Zhu M., Jin R. (2010). Thiolate-Protected Au*_n_* Nanoclusters as Catalysts for Selective Oxidation and Hydrogenation Processes. Adv. Mater..

[67] Zhu Y., Qian H., Jin R. (2010). An Atomic-Level Strategy for Unraveling Gold Nanocatalysis from the Perspective of Au*_n_*(SR)*_m_* Nanoclusters. Chem. Eur. J..

[68] Wang S., Jin S., Yang S., Chen S., Song Y., Zhang J., Zhu M. (2015). Total Structure Determination of Surface Doping [Ag_46_Au_24_(SR)_32_](BPh_4_)_2_ Nanocluster and Its Structure-Related Catalytic Property. Science Adv..

[69] Chai J., Chong H., Wang S., Yang S., Wu M., Zhu M. (2016). Controlling the Selectivity of Catalytic Oxidation of Styrene over Nanocluster Catalysts. RSC Adv..

[70] Turner M., Golovko V.B., Vaughan O.P.H., Abdulkin P., Berenguer-Murcia A., Tikhov M.S., Johnson B.F.G., Lambert R.M. (2008). Selective Oxidation with Dioxygen by Gold Nanoparticle Catalysts Derived from 55-Atom Clusters. Nature.

[71] Zhang B., Kaziz S., Li H., Hevia M.G., Wodka D., Mazet C., Bürgi T., Barrabés N. (2015). Modulation of Active Sites in Supported Au_38_(SC_2_H_4_Ph)_24_ Cluster Catalysts: Effect of Atmosphere and Support Material. J. Phys. Chem. C.

[72] Liu Y., Tsunoyama H., Akita T., Xie S., Tsukuda T. (2011). Aerobic Oxidation of Cyclohexane Catalyzed by Size-Controlled Au Clusters on Hydroxyapatite: Size Effect in the Sub-2 nm Regime. ACS Catal..

[73] Liu C., Yan C., Lin J., Yu C., Huang J., Li G. (2015). One-Pot Synthesis of Au_144_(SCH_2_Ph)_60_ Nanoclusters and Their Catalytic Application. J. Mater. Chem. A.

[74] Li G., Qian H., Jin R. (2012). Gold Nanocluster-Catalyzed Selective Oxidation of Sulfide to Sulfoxide. Nanoscale.

[75] Chen Y., Wang J., Liu C., Li Z., Li G. (2016). Kinetically Controlled Synthesis of Au_102_(SPh)_44_ Nanoclusters and Catalytic Application. Nanoscale.

[76] Kauffman D.R., Alfonso D., Matranga C., Ohodnicki P., Deng X., Siva R.C., Zeng C., Jin R. (2014). Probing Active Site Chemistry with Differently Charged Au_25_*^q^* Nanoclusters (*q* = −1, 0, +1). Chem. Sci..

[77] Kauffman D.R., Alfonso D., Matranga C., Qian H., Jin R. (2012). Experimental and Computational Investigation of Au_25_ Clusters and CO_2_: A Unique Interaction and Enhanced Electrocatalytic Activity. J. Am. Chem. Soc..

[78] Zhao S., Jin R., Jin R. (2018). Opportunities and Challenges in CO_2_ Reduction by Gold- and Silver-Based Electrocatalysts: From Bulk Metals to Nanoparticles and Atomically Precise Nanoclusters. ACS Energy Lett..

[79] Andrews E., Katla S., Kumar C., Patterson M., Sprunger P., Flake J. (2015). Electrocatalytic Reduction of CO_2_ at Au Nanoparticle Electrodes: Effects of Interfacial Chemistry on Reduction Behavior. J. Electrochem. Soc..

[80] Kauffman D.R., Thakkar J., Siva R., Matranga C., Ohodnicki P.R., Zeng C., Jin R. (2015). Efficient Electrochemical CO_2_ Conversion Powered by Renewable Energy. ACS Appl. Mater. Inter..

[81] Zhao S., Austin N., Li M., Song Y., House S.D., Bernhard S., Yang J.C., Mpourmpakis G., Jin R. (2018). Influence of Atomic-Level Morphology on Catalysis: The Case of Sphere and Rod-Like Gold Nanoclusters for CO_2_ Electroreduction. ACS Catal..

[82] Jupally V.R., Dharmaratne A.C., Crasto D., Huckaba A.J., Kumara C., Nimmala P.R., Kothalawala N., Delcamp J.H., Dass A. (2014). Au_137_(SR)_56_ Nanomolecules: Composition, Optical Spectroscopy, Electrochemistry and Electrocatalytic Reduction of CO_2_. Chem. Commun..

[83] Alfonso D.R., Kauffman D., Matranga C. (2016). Active Sites of Ligand-Protected Au_25_ Nanoparticle Catalysts for CO_2_ Electroreduction to CO. J. Chem. Phys..

[84] Seh Z.W., Kibsgaard J., Dickens C.F., Chorkendorff I., Nørskov J.K., Jaramillo T.F. (2017). Combining Theory and Experiment in Electrocatalysis: Insights into Materials Design. Science.

[85] Jiao Y., Zheng Y., Jaroniec M., Qiao S.Z. (2015). Design of Electrocatalysts for Oxygen- and Hydrogen-Involving Energy Conversion Reactions. Chem. Soc. Rev..

[86] Sabatier P. (1911). Hydrogénations Et Déshydrogénations Par Catalyse. Ber. Dtsch. Chem. Ges..

[87] Parsons R. (1958). The Rate of Electrolytic Hydrogen Evolution and the Heat of Adsorption of Hydrogen. Trans. Faraday Soc..

[88] Benck J.D., Hellstern T.R., Kibsgaard J., Chakthranont P., Jaramillo T.F. (2014). Catalyzing the Hydrogen Evolution Reaction (HER) with Molybdenum Sulfide Nanomaterials. ACS Catal..

[89] Jaramillo T.F., Jørgensen K.P., Bonde J., Nielsen J.H., Horch S., Chorkendorff I. (2007). Identification of Active Edge Sites for Electrochemical H_2_ Evolution from MoS_2_ Nanocatalysts. Science.

[90] Cao B., Veith G.M., Neuefeind J.C., Adzic R.R., Khalifah P.G. (2013). Mixed Close-Packed Cobalt Molybdenum Nitrides as Non-Noble Metal Electrocatalysts for the Hydrogen Evolution Reaction. J. Am. Chem. Soc..

[91] Popczun E.J., McKone J.R., Read C.G., Biacchi A.J., Wiltrout A.M., Lewis N.S., Schaak R.E. (2013). Nanostructured Nickel Phosphide as an Electrocatalyst for the Hydrogen Evolution Reaction. J. Am. Chem. Soc..

[92] Kibler L.A., El-Aziz A.M., Hoyer R., Kolb D.M. (2005). Tuning Reaction Rates by Lateral Strain in a Palladium Monolayer. Angew. Chem. Int. Ed..

[93] McCrory C.C.L., Jung S., Peters J.C., Jaramillo T.F. (2013). Benchmarking Heterogeneous Electrocatalysts for the Oxygen Evolution Reaction. J. Am. Chem. Soc..

[94] Stoerzinger K.A., Qiao L., Biegalski M.D., Shao-Horn Y. (2014). Orientation-Dependent Oxygen Evolution Activities of Rutile IrO_2_ and RuO_2_. J. Phys. Chem. Lett..

[95] Zhang B., Zheng X., Voznyy O., Comin R., Bajdich M., García-Melchor M., Han L., Xu J., Liu M., Zheng L. (2016). Homogeneously Dispersed Multimetal Oxygen-Evolving Catalysts. Science.

[96] Gasteiger H.A., Kocha S.S., Sompalli B., Wagner F.T. (2005). Activity Benchmarks and Requirements for Pt, Pt-Alloy, and Non-Pt Oxygen Reduction Catalysts for PEMFCs. Appl. Catal. B.

[97] Gasteiger H.A., Marković N.M. (2009). Just a Dream−or Future Reality?. Science.

[98] Siahrostami S., Verdaguer-Casadevall A., Karamad M., Deiana D., Malacrida P., Wickman B., Escudero-Escribano M., Paoli E.A., Frydendal R., Hansen T.W. (2013). Enabling Direct H_2_O_2_ Production through Rational Electrocatalyst Design. Nat. Mater..

[99] Nørskov J.K., Rossmeisl J., Logadottir A., Lindqvist L., Kitchin J.R., Bligaard T., Jónsson H. (2004). Origin of the Overpotential for Oxygen Reduction at a Fuel-Cell Cathode. J. Phys. Chem. B.

[100] Huang X., Zhao Z., Cao L., Chen Y., Zhu E., Lin Z., Li M., Yan A., Zettl A., Wang Y.M. (2015). High-Performance Transition Metal–Doped Pt_3_Ni Octahedra for Oxygen Reduction Reaction. Science.

[101] Peng Z., Yang H. (2009). Synthesis and Oxygen Reduction Electrocatalytic Property of Pt-on-Pd Bimetallic Heteronanostructures. J. Am. Chem. Soc..

[102] Kwak K., Choi W., Tang Q., Kim M., Lee Y., Jiang D.-e., Lee D. (2017). A Molecule-Like PtAu_24_(SC_6_H_13_)_18_ Nanocluster as an Electrocatalyst for Hydrogen Production. Nat. Commun..

[103] Choi W., Hu G., Kwak K., Kim M., Jiang D.-e., Choi J.-P., Lee D. (2018). Effects of Metal-Doping on Hydrogen Evolution Reaction Catalyzed by MAu_24_ and M_2_Au_36_ Nanoclusters (M = Pt, Pd). ACS Appl. Mater. Inter..

[104] Kwak K., Lee D. (2019). Electrochemistry of Atomically Precise Metal Nanoclusters. Acc. Chem. Res..

[105] Hu G., Tang Q., Lee D., Wu Z., Jiang D.-e. (2017). Metallic Hydrogen in Atomically Precise Gold Nanoclusters. Chem. Mater..

[106] Du Y., Xiang J., Ni K., Yun Y., Sun G., Yuan X., Sheng H., Zhu Y., Zhu M. (2018). Design of Atomically Precise Au_2_Pd_6_ Nanoclusters for Boosting Electrocatalytic Hydrogen Evolution on MoS_2_. Inorg. Chem. Front..

[107] Eguchi D., Sakamoto M., Teranishi T. (2018). Ligand Effect on the Catalytic Activity of Porphyrin-Protected Gold Clusters in the Electrochemical Hydrogen Evolution Reaction. Chem. Sci..

[108] Zhao S., Jin R., Song Y., Zhang H., House S.D., Yang J.C., Jin R. (2017). Atomically Precise Gold Nanoclusters Accelerate Hydrogen Evolution over MoS_2_ Nanosheets: The Dual Interfacial Effect. Small.

[109] Kwak K., Choi W., Tang Q., Jiang D.-e., Lee D. (2018). Rationally Designed Metal Nanocluster for Electrocatalytic Hydrogen Production from Water. J. Mater. Chem. A.

[110] Zhao S., Jin R., Abroshan H., Zeng C., Zhang H., House S.D., Gottlieb E., Kim H.J., Yang J.C., Jin R. (2017). Gold Nanoclusters Promote Electrocatalytic Water Oxidation at the Nanocluster/CoSe_2_ Interface. J. Am. Chem. Soc..

[111] Chen W., Chen S. (2009). Oxygen Electroreduction Catalyzed by Gold Nanoclusters: Strong Core Size Effects. Angew. Chem. Int. Ed..

[112] Wang L., Tang Z., Yan W., Yang H., Wang Q., Chen S. (2016). Porous Carbon-Supported Gold Nanoparticles for Oxygen Reduction Reaction: Effects of Nanoparticle Size. ACS Appl. Mater. Inter..

[113] Sumner L., Sakthivel N.A., Schrock H., Artyushkova K., Dass A., Chakraborty S. (2018). Electrocatalytic Oxygen Reduction Activities of Thiol-Protected Nanomolecules Ranging in Size from Au_28_(SR)_20_ to Au_279_(SR)_84_. J. Phys. Chem. C.

[114] Jones T.C., Sumner L., Ramakrishna G., Hatshan M.b., Abuhagr A., Chakraborty S., Dass A. (2018). Bulky *t*-Butyl Thiolated Gold Nanomolecular Series: Synthesis, Characterization, Optical Properties, and Electrocatalysis. J. Phys. Chem. C.

[115] Lu Y., Jiang Y., Gao X., Chen W. (2014). Charge State-Dependent Catalytic Activity of [Au_25_(SC_12_H_25_)_18_] Nanoclusters for the Two-Electron Reduction of Dioxygen to Hydrogen Peroxide. Chem. Commun..

[116] Kwak K., Azad U.P., Choi W., Pyo K., Jang M., Lee D. (2016). Efficient Oxygen Reduction Electrocatalysts Based on Gold Nanocluster–Graphene Composites. ChemElectroChem.

[117] Skúlason E., Tripkovic V., Björketun M.E., Gudmundsdóttir S., Karlberg G., Rossmeisl J., Bligaard T., Jónsson H., Nørskov J.K. (2010). Modeling the Electrochemical Hydrogen Oxidation and Evolution Reactions on the Basis of Density Functional Theory Calculations. J. Phys. Chem. C.

[118] Sakamoto M., Tanaka D., Tsunoyama H., Tsukuda T., Minagawa Y., Majima Y., Teranishi T. (2012). Platonic Hexahedron Composed of Six Organic Faces with an Inscribed Au Cluster. J. Am. Chem. Soc..

[119] Tanaka D., Inuta Y., Sakamoto M., Furube A., Haruta M., So Y.-G., Kimoto K., Hamada I., Teranishi T. (2014). Strongest Π–Metal Orbital Coupling in a Porphyrin/Gold Cluster System. Chem. Sci..

[120] Li P., Wang M., Duan X., Zheng L., Cheng X., Zhang Y., Kuang Y., Li Y., Ma Q., Feng Z. (2019). Boosting Oxygen Evolution of Single-Atomic Ruthenium through Electronic Coupling with Cobalt-Iron Layered Double Hydroxides. Nat. Commun..

[121] Lee Y., Suntivich J., May K.J., Perry E.E., Shao-Horn Y. (2012). Synthesis and Activities of Rutile IrO_2_ and RuO_2_ Nanoparticles for Oxygen Evolution in Acid and Alkaline Solutions. J. Phys. Chem. Lett..

[122] Mattioli G., Giannozzi P., Amore Bonapasta A., Guidoni L. (2013). Reaction Pathways for Oxygen Evolution Promoted by Cobalt Catalyst. J. Am. Chem. Soc..

[123] Frydendal R., Paoli E.A., Knudsen B.P., Wickman B., Malacrida P., Stephens I.E.L., Chorkendorff I. (2014). Benchmarking the Stability of Oxygen Evolution Reaction Catalysts: The Importance of Monitoring Mass Losses. ChemElectroChem.

[124] Zhuang Z., Sheng W., Yan Y. (2014). Synthesis of Monodispere Au@Co_3_O_4_ Core-Shell Nanocrystals and Their Enhanced Catalytic Activity for Oxygen Evolution Reaction. Adv. Mater..

[125] Zhao X., Gao P., Yan Y., Li X., Xing Y., Li H., Peng Z., Yang J., Zeng J. (2017). Gold Atom-Decorated CoSe_2_ Nanobelts with Engineered Active Sites for Enhanced Oxygen Evolution. J. Mater. Chem. A.

[126] Li Z.-y., Ye K.-h., Zhong Q.-s., Zhang C.-j., Shi S.-t., Xu C.-w. (2014). Au–Co_3_O_4_/C as an Efficient Electrocatalyst for the Oxygen Evolution Reaction. ChemPlusChem.

[127] Mills G., Gordon M.S., Metiu H. (2003). Oxygen Adsorption on Au Clusters and a Rough Au(111) Surface: The Role of Surface Flatness, Electron Confinement, Excess Electrons, and Band Gap. J. Chem. Phys..

[128] Okumura M., Kitagawa Y., Kawakami T., Haruta M. (2008). Theoretical Investigation of the Hetero-Junction Effect in PVP-Stabilized Au_13_ Clusters. The Role of PVP in Their Catalytic Activities. Chem. Phys. Lett..

[129] Yin H., Tang H., Wang D., Gao Y., Tang Z. (2012). Facile Synthesis of Surfactant-Free Au Cluster/Graphene Hybrids for High-Performance Oxygen Reduction Reaction. ACS Nano.

